# Influence of Different Shellfish Matrices on the Separation of PSP Toxins Using a Postcolumn Oxidation Liquid Chromatography Method

**DOI:** 10.3390/toxins7041324

**Published:** 2015-04-15

**Authors:** Verónica Rey, Amparo Alfonso, Luis M. Botana, Ana M. Botana

**Affiliations:** 1 Department of Analytical Chemistry, Science Faculty, University of Santiago de Compostela, Lugo 27002, Spain; E-Mail: veronica.rey@rai.usc.es; 2 Department of Pharmacology, Veterinary Faculty, University of Santiago de Compostela, Lugo 27002, Spain; E-Mail: amparo.alfonso@usc.es

**Keywords:** paralytic shellfish poisoning, toxins, postcolumn oxidation method, interfering matrix peaks

## Abstract

The separation of PSP toxins using liquid chromatography with a post-column oxidation fluorescence detection method was performed with different matrices. The separation of PSP toxins depends on several factors, and it is crucial to take into account the presence of interfering matrix peaks to produce a good separation. The matrix peaks are not always the same, which is a significant issue when it comes to producing good, reliable results regarding resolution and toxicity information. Different real shellfish matrices (mussel, scallop, clam and oyster) were studied, and it was seen that the interference is not the same for each individual matrix. It also depends on the species, sampling location and the date of collection. It was proposed that separation should be accomplished taking into account the type of matrix, as well as the concentration of heptane sulfonate in both solvents, since the mobile phase varies regarding the matrix. Scallop and oyster matrices needed a decrease in the concentration of heptane sulfonate to separate GTX4 from matrix peaks, as well as dcGTX3 for oysters, with a concentration of 6.5 mM for solvent A and 6.25 mM for solvent B. For mussel and clam matrices, interfering peaks are not as large as they are in the other group, and the heptane sulfonate concentration was 8.25 mM for both solvents. Also, for scallops and oysters, matrix interferences depend not only on the sampling site but also on the date of collection as well as the species; for mussels and clams, differences are noted only when the sampling site varies.

## 1. Introduction

Paralytic shellfish toxins are potent compounds produced by several species of dinoflagellates, such as *Alexandrium tamarense*, *Gymnodinium catenatum* and *Pyrodinium bahamense*, and ingestion of contaminated shellfish with PSP toxins can cause serious life-threatening intoxications [1]. The highly toxic and unpredictable nature of these biotoxin blooms means that the toxin content of shellfish in affected areas is monitored [2]. PSP monitoring programs rely on relatively intensive sampling and analysis protocols that require rapid, sensitive, accurate, and precise analytical techniques for the analysis of PSP toxins [3].

The mouse bioassay [4] is the method traditionally used to determine the presence of PSP toxins in shellfish. However, ethical considerations regarding the use of mammals in assays led to a search for other methods, and high-performance liquid chromatography (HPLC) with fluorimetric detection was chosen [5,6].

High-performance liquid chromatography methods are widely used to identify and quantify PSP toxins present in seafood, and they can also establish the toxin's profile, which can be very different depending on the geographical area, the phytoplankton species and the shellfish species [7,8]. HPLC-FLD with a precolumn oxidation method, also called the Lawrence method, was validated by the Association of Official Analytical Chemists through a collaborative study [9] and was integrated into European Directives to act as a legal alternative to the mouse bioassay [10] for the determination of PSP toxins.

In the last few years, considerable progress has been made in the development of these methods as an alternative to bioassay [11,12,13]. In 2012, Turner *et al*. [14] proposed a refinement of AOAC method 2005.06 for the determination of PSP toxins in oysters, because in this matrix the toxin recovery and sensitivity was poor. The refined LC-FLD method improved performance characteristics for the determination of PSP toxins in whole king and queen scallops. However, the lack of standards (C3, C4, GTX6, dcGTX1, dcGTX4), for the correct identification and quantitation of PSP toxins was a handicap, as was the inability to distinguish between certain analogues with different toxicity; all this creates problems with the adequate identification and quantification of several PSP toxins [8].

Along with the precolumn method, a postcolumn oxidation HPLC-FLD method proposed by Oshima [15,16] has been used for years to detect and quantify PSP toxins. This postcolumn oxidation (PCOX) method has undergone several modifications [17], and was used successfully in a collaborative study [18], which greatly increased its relevance; it then became an official AOAC method [19], and was proposed to replace the current AOAC mouse bioassay as well, as an alternative to the precolumn method. Nevertheless, there are some studies which show that the analysis of PSP toxins is different regarding the sample matrix; Turner *et al*. [12] reported the possibility of fluorescence enhancement of PSP toxins in the oyster matrix, although there is no evidence for matrix components falsely enhancing the toxin signals. They pointed out that it is possible that variations in fluorescence enhancement or suppression previously observed between different shellfish species may also occur between different samples of the same species with different spatial and temporal origins.

There is some evidence for the potential effect of high concentrations of zinc and manganese in oyster resulting in a suppression of the MBA (mouse bioassay) response [14]; moreover, it is noted that whilst high concentrations of zinc do not affect the precolumn oxidation method, there is a potential for metal ions to interfere with the ion-pairing-based postcolumn oxidation method [20].

All this shows that there are differences between different matrices and also between either pre- or post-column methods. With this work, several samples from different shellfish matrices were analyzed using the validated postcolumn method. The differences in separation regarding the seafood matrix were studied. It is shown that not all the matrices have the same profile; it was necessary to change the conditions of the method when the sample matrix was changed.

## 2. Results

The initial PCOX method underwent several changes: postcolumn conditions were changed (oxidant flow, acid flow and acid concentration) to reach a pH outflow of 5–7 [19].

The reaction temperature was tested from 65 °C to 85 °C in the water bath [16]. It has been seen that, up to 80 °C, the higher the temperature, the bigger the peak area signal in the chromatograms, but from 85 °C some peaks were lower. Therefore, the working reaction temperature was 80 °C.

The shellfish tissue extract used for diluting GTXs and STXs was prepared with minor modifications. It was found that when adding 35 µL of 1 M NaOH to reach pH = 3, as recommended after deproteination, GTX1, GTX4 and NEO degraded after successive injections. Generally, the PSP toxins are stable under acidic conditions, although the stability depends on the chemical structure; GTX1, GTX4 and NEO are less stable at acidic pH than GTX2, GTX3 and STX [21]. Therefore, pH values of 4 and 5 were tested observing that at pH = 4, toxins were more stable and the signal was greater.

**Figure 1:**
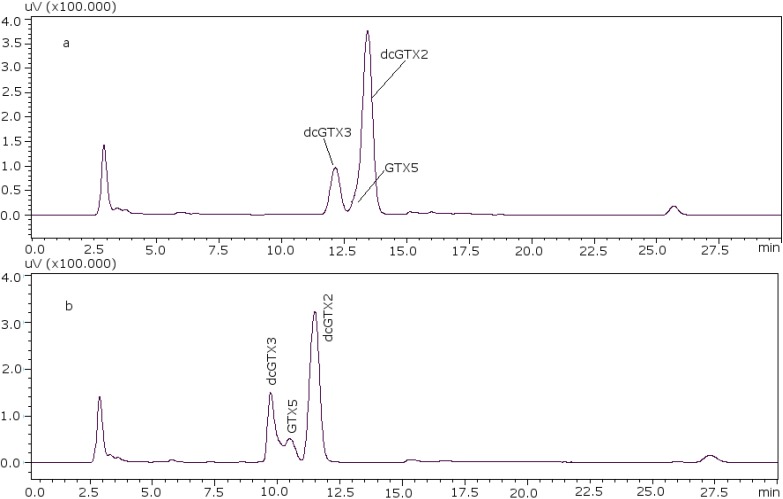
Chromatographic separation of dcGTX3-GTX5-dcGTX2, (**a**) with 11 mM heptane sulfonate in mobile phase; (**b**) with 8.25 mM heptane sulfonate in mobile phase.

**Figure 2:**
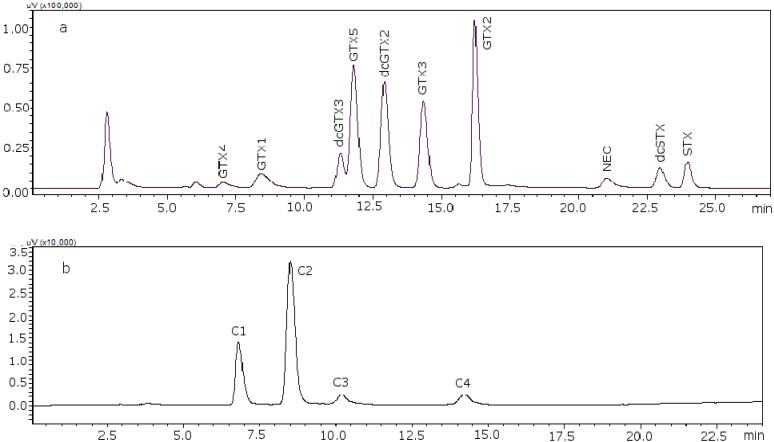
Chromatograms of the two working standard solutions. (**a**) Separation of the gonyautoxins and saxitoxins in mussel tissue using 8.25 mM heptane sulfonate; (**b**) separation of C-toxins in DIW.

The age/status of the LC columns has a large impact on the resolution, and the pH of the mobile phase as well as the concentration of the reagents in it are also crucial. The most important component is the ion-pair reagent heptane sulfonate; in our laboratory a better separation was obtained when the concentration of heptane sulfonate was adjusted to 8.25 mM [22]. With these conditions it was possible to separate GTX5 from dcGTX2, as it is shown in Figure 1a (11 mM heptane sulfonate) and Figure 1b (8.25 mM heptane sulfonate). Figure 2 shows the chromatograms of the two working standard solutions after checking how new conditions work for all the standards, where GTXs and STXs are separated in mussel tissue (Figure 2a) and Cs are separated in deionized water (DIW) (Figure 2b).

Routine analysis of samples require the use of matrix-fortified standards to diminish the effect that the matrix has on the retention time of the early eluting toxins; however, sometimes these matrix peaks elute with the toxins and so it is important to get optimal elution conditions to isolate these interfering peaks at the maximum and achieve a good resolution. Therefore, the effect of different toxin-free matrices on the separation of PSP toxins was studied. Mussel, clam, scallop and oyster matrices were used. Commercial shellfish samples were used in all cases and not toxin-free homogenates like NRCC-CRM-Zero-Mus [18], because the behavior of real matrices could be observed.

**Figure 3:**
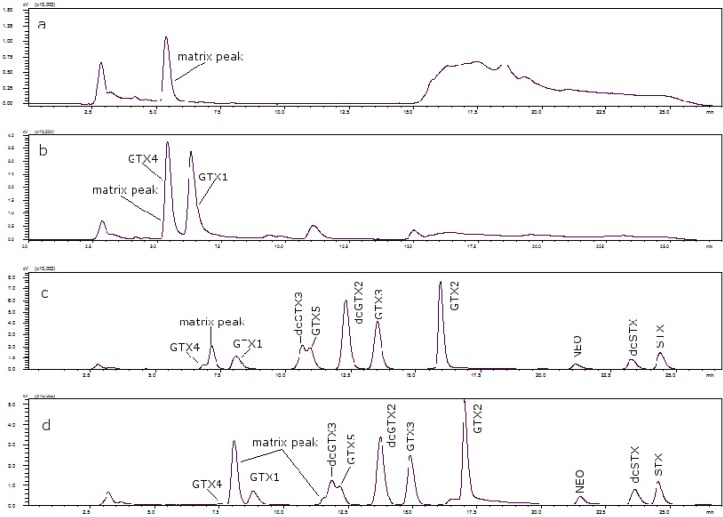
(**a**) Scallop PSP toxin-free with 11 mM heptane sulfonate in mobile phase; (**b**) GTX4 and GTX1 in scallop tissue with 11 mM heptane sulfonate in mobile phase; (**c**) PSP toxins standards in scallop tissue with 6.5 mM heptane sulfonate in solvent A and 6.25 mM heptane sulfonate in solvent B; (**d**) PSP toxins standards in oyster tissue with 6.5 mM heptane sulfonate in solvent A and 6.25 mM heptane sulfonate in solvent B.

It was found that the behavior of PSP toxins in mussels and clams is similar. The same conditions were used for these two shellfish. As mentioned, the concentration of heptane sulfonate for both solvents in order to get a good resolution was 8.25 mM in all cases. However, one scallop matrix peak coeluted with GTX4; to separate them heptane sulfonate concentration was changed in solvent A, with 6.5 mM being the appropriate concentration. When oyster was studied the same problem was found as in the case of the scallop, namely that GTX4 coeluted with a matrix peak and also another peak coeluted with dcGTX3. Therefore, the heptane sulfonate concentration was modified to 6.5 mM in solvent A and to 6.25 mM in solvent B, although the separation between dcGTX3 and the matrix peak was not optimal. Figure 3 shows the chromatograms of (a) a toxin-free scallop tissue, where it is possible to see the matrix peak for a 11 mM heptane sulfonate concentration; (b) the overlapping of GTX4 and that peak at that concentration; and finally, (c) the separation of both peaks, when the concentration of heptane sulfonate was changed. The chromatogram in (d) shows the separation obtained for oyster.

After optimization of separation conditions, the linearity, repeatability, LODs and LOQs were studied. The linearity results are summarized in Table 1, and all the linear correlation coefficients are higher than 0.98, showing an excellent correlation. The lowest value for mussels was 0.0002 mg/kg and the highest value was 5.2756 mg/kg; for clams they were 0.0026 mg/kg and 0.5284 mg/kg, respectively; for scallops they were 0.0006 mg/kg and 0.8220 mg/kg; and for oysters, the lowest and highest values were 0.0171 mg/kg and 4.1170 mg/kg, respectively.

**Table 1:** Linearity of the instrument response to each matrix-fortified PSP standard (mg/kg).

Toxin	Matrix	Linearity range	Calibration curve	
			Equation	Correlation (R^2^)
GTX4	Mussel	0.0004–0.1341	*y* = 472166*x* − 1281	0.9823
	Clam	0.0042–0.2681	*y* = 407086*x* + 218418	0.9851
	Scallop	0.0010–0.2681	*y* = 175936*x* − 2349	0.9904
	Oyster	0.0495–0.7922	*y* = 225692*x* − 914	0.9989
GTX1	Mussel	0.0064–1.6442	*y* = 275537*x* + 29094	0.9905
	Clam	0.0128–0.4110	*y* = 104377*x* + 32765	0.9855
	Scallop	0.0032–0.8220	*y* = 266520*x* − 10630	0.9929
	Oyster	0.1518–1.2141	*y* = 142355*x* + 1477	0.9978
dcGTX3	Mussel	0.0002–0.2255	*y* = 3 × 10^6^*x* + 37075	0.9917
	Clam	0.0046–0.1480	*y* = 2 × 10^6^*x* − 14452	0.9779
	Scallop	0.0006–0.1480	*y* = 2 × 10^6^*x* − 19014	0.9927
	Oyster	0.0185–0.2980	*y* = 737742*x* + 6381	0.9879
GTX5	Mussel	0.0004–1.0620	*y* = 105632*x* − 2213	0.9983
	Clam	0.0083–0.2655	*y* = 295206*x* − 94	0.9816
	Scallop	0.0021–0.5310	*y* = 510734*x* − 14907	0.9938
	Oyster	0.0576–0.9222	*y* = 213243*x* + 5600	0.9949
dcGTX2	Mussel	0.0008–0.8032	*y* = 3 × 10^6^*x* + 18524	0.9976
	Clam	0.0165–0.5284	*y* = 1 × 10^6^*x* − 80684	0.9800
	Scallop	0.0021–0.5284	*y* = 2 × 10^6^*x* − 44732	0.9947
	Oyster	0.0332–2.1289	*y* = 424621*x* + 5576	0.9866
GTX3	Mussel	0.0002–0.3421	*y* = 3 × 10^6^*x* + 69830	0.9879
	Clam	0.0027–0.0428	*y* = 1 × 10^6^*x* + 5344	0.9904
	Scallop	0.0007–0.1711	*y* = 4 × 10^6^*x* − 27177	0.9960
	Oyster	0.0171–1.0941	*y* = 758162*x* − 4082	0.9989
GTX2	Mussel	0.0005–1.0296	*y* = 2 × 10^6^*x* + 74858	0.9943
	Clam	0.0080–0.2574	*y* = 823267*x* − 20717	0.9801
	Scallop	0.0020–0.5148	*y* = 2 × 10^6^*x* − 26429	0.9969
	Oyster	0.0514–1.6464	*y* = 497495*x* − 3098	0.9877
NEO	Mussel	0.0032–0.8186	*y* = 157597*x* − 4730	0.9972
	Clam	0.0064–0.1023	*y* = 57166*x* + 1369	0.9892
	Scallop	0.0016–0.4093	*y* = 169005*x* − 6014	0.9907
	Oyster	0.2573–4.1170	*y* = 54360*x* + 1380	0.9981
dcSTX	Mussel	0.0013–0.3281	*y* = 412156*x* − 4413	0.9970
	Clam	0.0026–0.0820	*y* = 462063*x* − 2023	0.9918
	Scallop	0.0006–0.0820	*y* = 522829*x* − 3457	0.9913
	Oyster	0.0390–2.4978	*y* = 127331*x* + 429	0.9943
STX	Mussel	0.0018–0.1183	*y* = 700921*x* + 339	0.9953
	Clam	0.0037–0.1183	*y* = 467605*x* − 3866	0.9850
	Scallop	0.0009–0.2367	*y* = 856619*x* − 9104	0.9964
	Oyster	0.0267–0.8543	*y* = 265150*x* − 1806	0.9957
C1	Mussel	0.0103–5.2756	*y* = 2 × 10^6^*x* − 50438	0.9947
C2	Mussel	0.0316–1.6197	*y* = 3 × 10^6^*x* − 40838	0.9964

Repeatability was studied, with 5 daily injections over 3 days (*n* = 15), at an intermediate concentration (linear calibration interval) for each matrix. Data for levels of concentration and %RSD are shown in Table 2; the %RSD for the toxins in each matrix is within the acceptable range (in most cases below 7%), and so the repeatability for all toxins in all matrixes appears to be consistent.

**Table 2:** Method repeatability, %RSD.

Toxin	µM	mg STX eq/kg	Mussels	Clams	Scallops	Oysters
GTX4	0.0815	0.02202	6.55	7.39	6.68	4.63
GTX1	0.2498	0.09242	6.34	7.20	6.65	3.88
dcGTX3	0.0400	0.00561	3.88	7.89	8.78	9.52
GTX5	0.1425	0.00816	3.94	5.76	5.46	2.36
dcGTX2	0.1749	0.00419	3.46	5.81	3.52	5.53
GTX3	0.0541	0.01284	4.20	3.64	2.65	3.96
GTX2	0.1627	0.02175	0.25	4.69	3.28	5.83
NEO	0.1623	0.05584	1.89	6.32	7.79	3.22
dcSTX	0.0800	0.01528	1.28	6.89	5.66	10.47
STX	0.0795	0.02959	2.23	7.55	8.02	12.48
C1	0.3135	0.00070	6.18			
C2	0.0962	0.00345	5.19			

LOD (limit of detection) and LOQ (limit of quantitation) values were both calculated for each matrix analyzing 5 replicate extracts of a blank matrix, repeated over 6 days (*n* = 30). The baseline signal-to-noise ratio at the approximate retention time for all toxins was calculated. The LOD value was obtained, taking the noise response (area units) multiplied by 3, converted to µmol and expressed as mg STX eq/kg for each toxin, and the LOQ was calculated as LOD × 3 [23]. Table 3 summarizes LODs and LOQs for each toxin in each matrix. The results show that sensitivity is different for the different matrices, and the best results in general are those obtained for mussels.

**Table 3:** LODs and LOQs (mg STX∙diHCl eq/kg) for the method.

Toxin	Mussels		Clams		Scallops		Oysters	
	LOD	LOQ	LOD	LOQ	LOD	LOQ	LOD	LOQ
**GTX4**	0.0183	0.0549	0.0201	0.0603	0.0221	0.0663	0.048	0.144
**GTX1**	0.0642	0.1926	0.0320	0.0960	0.0488	0.1464	0.057	0.171
**dcGTX3**	0.0071	0.0213	0.0096	0.0288	0.0097	0.0291	0.003	0.009
**GTX5**	0.0011	0.0033	0.0088	0.0264	0.0298	0.0894	0.025	0.075
**dcGTX2**	0.0210	0.0630	0.0181	0.0543	0.0244	0.0732	0.030	0.090
**GTX3**	0.0055	0.0165	0.0072	0.0216	0.0073	0.0219	0.011	0.033
**GTX2**	0.0099	0.0297	0.0306	0.0918	0.0199	0.0597	0.023	0.069
**NEO**	0.0239	0.0717	0.0329	0.0987	0.0375	0.1125	0.018	0.054
**dcSTX**	0.0047	0.0141	0.0172	0.0516	0.0074	0.0222	0.0005	0.001
**STX**	0.0100	0.0300	0.0394	0.1182	0.0111	0.0333	0.009	0.027
**C1**	0.0001	0.0003						
**C2**	0.0011	0.0033						

In the post-column method, the standards were diluted firstly in DIW (pH = 5) for Cs toxins and in HCl 0.003 M for GTXs and STXs. New dilutions either in DIW (pH = 5) for Cs toxins or in shellfish extract for GTXs and STXs were prepared; the tissue used at this stage should be the matrix being analyzed at the moment in the laboratory, because the conditions (heptane sulfonate concentration in mobile phase) for each matrix are different, as mentioned.

**Figure 4:**
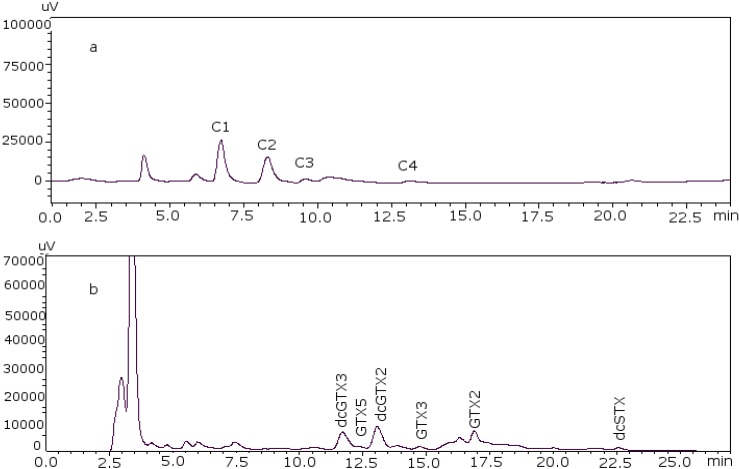
PSP toxin profile of sample PSP3. (**a**) C toxins and (**b**) GTX and STX toxins.

The next step was the analysis of PSP toxins in several samples using the post-column method to verify that the changes made were effective in real samples of different species, different origins and harvesting date. Toxin profiles are illustrated in Figure 4 for sample PSP3 (clam), as an example, and the results obtained for the quantification of each PSP toxin in the samples are summarized in Table 4.

**Table 4:** PSP toxin concentrations (µg/kg) in the samples obtained using HPLC with the post-column method.

µg/kg	GTX 1	GTX4	dcGTX2	dcGTX3	GTX5	GTX2	GTX3	NEO	dcSTX	STX	C1	C2	C3	C4
**PSP5**	73.6	245.5			2.9	14.6	14.5						-	-
**EXT.2**	65.5	43.9				NQ *	10.6							
**PSP6**	148	237.4	4.1	NQ *	3.3	20.1	24.6						-	-
**EXT.7**	13.5	515.2	4.4	NQ *		14.7	14							
**PSP4**	624.7	176.5	4.1	2.9		15.2	11.8				35.9	19.4	-	-
**EXT.1**	34.5	NQ *	4.1	2.5		0.01	12				134.9	47.3		
**PSP2**			33.3	10.3							59.1	44.5	-	-
**PSP3**			82.3	45.3	55.2	58.9	22.2		34.8		175.7	73.1	-	-
**PSP1**	68.9	ND *	54.4	56.3					43.9				-	-
**EXT.3**	45.4	56.2	5.7	5.4		11.6	10.4				149.2	122.6		
**EXT.6**	157.7	264.1	NQ *	NQ *		12.3	16.4				139.1	53.7		
**EXT.8**	72.93	156.2	4.2	NQ *		17.6	12.9							
**EXT.9**														
**PSP7**			122.7	44.2		121.9	51.2		309.6				-	-
**PSP8**	162.8	NQ *	917.4	482.6		103.9	47.4		627.5	17.7				
**PSP11**	57.6	184.9			4.3	17.9	1.4						-	-
**PSP12**			93.1	ND *		422.9	193.5		64.2	14.6				
**PSP10**			167.4	187.2	234.3	213.6	203.5		781.7		11.2	3.4	-	-
**EXT.4**			131.4	165.5	147.6	161.8	169.2		386.3		57.3	32.3		
**PSP 10H**			1934.2	4265.3	343.4	30	441.9		469.5	282.3	14.4	5.5		
**PSP 10G**			92.3	215.7	150.2	72.7	59.9		377.5				-	-
**PSP 10B**			66.64	158.64	94.49				140.23				-	-
**PSP 10M**			59.44	63.87	120.18	26.35	51.67		185.3				-	-
**PSP 10Ma**			37.83	87.3	148.89				135.49				-	-
**PSP 13**	30.70	2.57	NQ *	14.97	3.96	23.04	35.86	27.36		5.62	17.55	8.10	-	-
**PSP 14**				26.44										
**PSP 15**							6.31							

* ND: not detected; NQ: not quantitated.

## 3. Discussion

This work was done to identify the PSP toxin profile of several samples with different geographical locations received from 2007 to 2013, using the post-column oxidation method. This method, validated by van de Riet *et al*. [18], improved on Oshima’s [16], which initially became quite popular because it could chromatographically separate all PSP toxins, although its main drawback is that it is a time-consuming method. With Van de Riet’s improvement, all individual PSP analogues are determined, except some metabolites, in a shorter time.

GTX1 and GTX4 are two key toxins in the method [18]. The fact that they are the earliest eluting analytes increases the chance that the presence of sample matrix peaks will alter the retention time of the compounds compared to what is seen in matrix-free standard solutions. The use of matrix-matched calibration solutions was implemented in an effort to combat this shift and to make the data interpretation easier. Therefore, it is a crucial point to separate and correctly identify the peaks due to the matrix. In a previous collaborative study [18], some laboratories had difficulties to separate an early eluting peak from GTX4. Moreover its high toxicity relative to STX gives as a result a significant effect on total toxicity, which makes critical to obtain GTX4 well resolved from any interfering peak.

**Figure 5:**
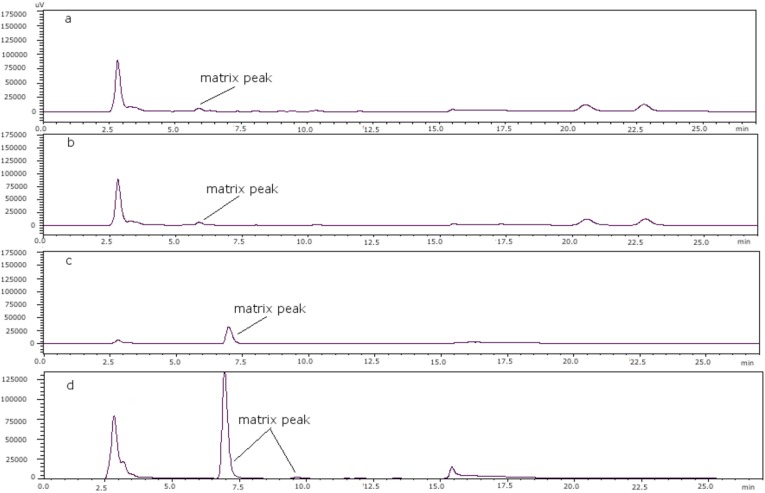
(**a**) Mussel PSP toxin-free; (**b**) clam PSP toxin-free; (**c**) scallop PSP toxin-free; (**d**) oyster PSP toxin-free.

The retention times of GTX1 and GTX4 in scallop samples were longer than those in other matrices [22], and the effect was dependent on sample loading; it is therefore reasonable to assume that there are other components in scallop extracts that elute in that region of the chromatogram, which disrupt the chromatographic equilibrium of components and cause a shift in retention times. However in this case it was found that these matrix components in scallops overlapped with GTX4, and the conditions described by Li *et al*. [22] did not work in our scallop samples, although they were appropriate for both mussels and clams. Figure 5 shows the toxin-free chromatograms for each of the matrices under study, after modifying the concentrations of heptane sulfonate, namely mussels, clams, scallops and oysters. In the cases of both scallops and oysters, a matrix peak at ca. 7.5 min is observed, and this is where GTX4 is eluted, so the eluting conditions had to be changed. When the concentration of heptane sulfonate was diminished in both solvents, separation was improved: this might be due to the role of heptane sulfonate, and it can be assumed that the interfering matrix peaks exhibit different behavior with it. As the optimal concentration of the ion-pair reagent is difficult to predict, it is empirically calculated in each case. For high-molecular weight counterions, a concentration of 5 × 10^−3^ M is often used [20], so the ion-pair is formed and the resulting non-charged complex is more easily attracted to the stationary phase. The heptane sulfonate concentrations used in this work are in all cases higher than 5 × 10^−3^ M, and although the utilization of primarily aqueous mobile phases and the use of an ion-pair reagent can be the causes of a phase malfunctioning in the column, while taking some precautions with the column toxins, separation can be accomplished.

**Figure 6:**
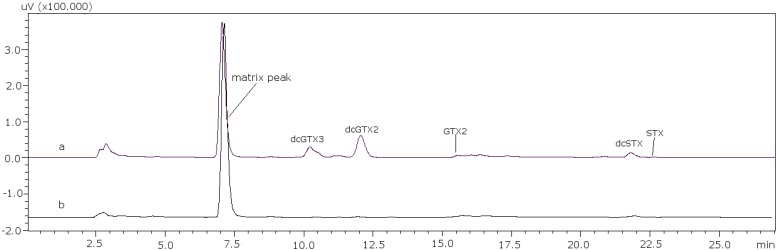
Overlaid chromatographic separation, showing temporal variations for: (a) Sample PSP8 and (b) sample PSP7.

In the analyzed scallop samples, this matrix peak was higher than in the scallops used as blanks, and this might be because they are different species. However, when comparing scallop samples which belong to the same species, it is observed that there are also differences. This is the case for samples PSP7 and PSP8; both are from the same area with a sampling difference of two months, and it was observed that the peaks due to the matrix are different despite being from the same area, so it seems that temporal variation is an important factor. Figure 6 shows the chromatograms of both samples, where the matrix peak in (a) (sample PSP8) is lower than in (b) (sample PSP7) and in this case that peak is the reason why the toxins are not seen in the scale shown in the chromatograms. When comparing samples from different areas it was seen that they also give different matrix peaks, so these differences in matrix peaks depend on the species, date of collection and geographical area. The reason might be that at different moments the marine streams and sea temperatures are different, which influences the presence of microorganisms and then algae blooms.

In oysters the same happens as in scallops for GTX1 and GTX4, but there is also a matrix peak which interferes with dcGTX3 (at ca. 11.6 min), so conditions were modified for this matrix. The best conditions are also those for scallops, which means diminishing the concentration of heptane sulfonate. It seems that there is a relationship between the presence of some matrix compounds in scallops and oysters and the decrease in the ion-pair reagent concentration. In the case of oysters, there was no sample available to study temporal and geographic variations.

For dcGTX3 there is another factor to take into account: the resolution related to GTX5 (Figure 1b), which must be at least 40% baseline-resolved between dcGTX3 and dcGTX2 [24]. When the column loses resolution, this toxin is not separated from dcGTX3, and this is the first indicator of the efficiency loss in the column. This factor adds more relevance to the importance of properly adjusting the concentration of heptane sulfonate to eliminate undesirable peaks.

Clam and mussel matrix peaks are not much larger problems than those of scallop and oyster because they are smaller and the retention time in each case is slightly shorter, around 6 min., which facilitates resolution. In this case the matrix peak in the blank is higher than those found in clam samples. Comparing samples from the same area and with the same date of collection, PSP2 and PSP3, shows they have exactly the same matrix peaks. If these samples are compared with PSP5, with a different collection date but from the same area, the results remain the same. Therefore, in the case of the same clam species with the same origin, the matrix peaks are equal: as the matrix peaks here are not so crucial, if samples from different origins are compared, such as PSP1, small differences are noted but not as significant as those in scallops. In the case of mussels, it is the same as with clams.

In summary, it is quite probable that sound analysis and quantification of toxins implies, first of all, looking at matrix peaks and deciding which concentration of heptane sulfonate is better with regard to the type of matrix.

Table 5 summarizes the heptane sulfonate concentration used for each matrix.

**Table 5:** Heptane sulfonate concentration for each matrix.

Heptane sulfonate concentration		
Type of matrix	Solvent A	Solvent B
**Mussel**	8.25 mM	8.25 mM
**Clam**	8.25 mM	8.25 mM
**Scallop**	6.5 mM	6.25 mM
**Oyster**	6.5 mM	6.25 mM

It has been observed that for the results obtained, the age and status of the column has a large impact on the resolution. This is due to working conditions that are not ideal for the column and therefore its short lifetime, and also the fact that when the column is not in use for some time this gets worse, meaning that if the column is stored and then recovered to analyze samples or standards, in some cases the column does not work at all, even if the amount of runs was not very high up to that moment. That is why it is important to leave the column with a small flow rate when not in use for some time. This will help in achieving a good resolution.

## 4. Experimental Section

### 4.1. Apparatus

Shimadzu LC system (Izasa Scientific, Madrid, Spain): degasser DGU-14A, LC-10A pumps, controller CBM-20A, fluorescence detector RF-10AXL, autoinjector with 70 vials and temperature controller SIL-20AC, column oven CTO-20AC. The software used is Shimadzu LC Solution.

Post-column reaction system: water bath at 80 °C and a homemade knitted reaction coil with a total volume of 1 mL (Supelco Sigma-Aldrich, Madrid, Spain). The oxidant is pumped through a Shimadzu LC-20AD pump and the acid through a Shimadzu LC-6A pump.

### 4.2. Chemicals and Solutions

All reagents are analytical grade or HPLC grade. Acetic acid, methanol, acetonitrile (MeCN), sodium hydroxide, periodic acid, hydrochloric acid 37%, ortho-phosphoric acid 85% and nitric acid 65% were from Panreac Quimica S.A. (Barcelona, Spain). Heptane sulfonate, trichloroacetic acid, tetrabutyl ammonium phosphate, ammonium hydroxide 28%–30% were purchased from Sigma-Aldrich (Madrid, Spain).

Saxitoxin (STX), neosaxitoxin (NEO), decarbamoylsaxitoxin (dcSTX), gonyautoxins 1 and 4 (GTX1, 4), gonyautoxins 2 and 3 (GTX2, 3), decarbamoylgonyautoxins 2 and 3 (dcGTX2, 3), GTX5 (B1), and C1 and C2 were provided by NRC (Institute for Marine Biosciences, Halifax, NS, Canada) for the identification of each toxin.

Algae sample containing C3 and C4 were provided by CIFGA (Lugo, Spain) and used as internal standards.

### 4.3. Samples Preparation

A total of 27 samples of 4 different species were analyzed. The shellfish species tested were mussels (*n* = 4), clams (*n* = 12), scallops (*n* = 10) and razor clams (*n* = 1).

Some of the samples were provided as extracts (EXT. X), which resulted from performing the MBA extraction [25], others were shellfish tissue homogenates (PSP X) and in this case the PCOX extraction was made. Table 6 shows samples information (code, species, date and area of collection).

**Table 6:** Samples analyzed information.

Sample	Code	Geographical origin	Date of collection
Clam	PSP 5	Area 1	19-01-2012
Clam	EXT. 2	Area 1	19-01-2012
Clam	PSP 6	Area 1	09-02-2012
Clam	EXT. 7	Area 1	09-02-2012
Clam	PSP 4	Area 1	19-01-2012
Clam	EXT. 1	Area 1	19-01-2012
Clam	PSP 2	Area 1	27-10-2011
Clam	PSP 3	Area 1	27-10-2011
Clam	PSP 1	Area 2	28-01-2008
Clam	EXT. 3	Area 1	27-10-2011
Clam	EXT. 6	Area 1	09-02-2012
Clam	EXT. 8	Area 1	09-02-2012
Mussel	EXT. 9	Area 3	-
Scallop	PSP 7	Area 4	10-06-2009
Scallop	PSP 8	Area 4	21-04-2009
Razor clam	PSP 11	Area 5	27-07-2007
Scallop	PSP 12	Area 5	07-06-2007
Scallop	PSP 10	Area 6	11-05-2012
Scallop	EXT. 4	Area 6	11-05-2012
Scallop (hepatopancreas)	PSP 10 H	Area 6	11-05-2012
Scallop (gonad)	PSP 10 G	Area 6	11-05-2012
Scallop (gills)	PSP 10 B	Area 6	11-05-2012
Scallop (muscle)	PSP 10 M	Area 6	11-05-2011
Scallop (mantle)	PSP 10 Ma	Area 6	11-05-2011
Mussel	PSP 13	Area 7	2013
Mussel	PSP 14	Area 8	2013
Mussel	PSP 15	Area 9	2013

PCOX extraction method involves 5.0 g of homogenized shellfish tissue transferred into a 50 mL polypropylene centrifuge tube, add 5.0 mL of 0.1 M HCl and close the tube and vortex to completely mix the contents. Check the pH of the mixture which should be between 2 and 4. If necessary, adjust the pH of the mixture by adding drops of 5 M HCl or 5 M NaOH while stirring the mixture. Place the tube into a boiling water bath and heat the sample for 5 min. Let cool down to room temperature [24]. Adjust, if necessary, the pH of the sample between 2 and 4. Centrifuge at 3000 × g for 10 min and decant the supernatant into a glass tube. Pipette 500 µL of the supernatant solution to a microcentrifuge tube and deproteinate by the addition of 25 µL 30% (*v/v*) trichloroacetic acid (TCA). Mix the contents using a vortex and then centrifuge at 16000 × g for 5 min. Add 40 µL of 1 M NaOH and centrifuge again. Filter the supernatant through a 0.2 µm nylon syringe filter into a vial for LC analysis. Inject aliquots (5–10 µL) into the system.

### 4.4. HPLC Analysis

The post-column oxidation and fluorescence detection method [18,19] was used with some modifications.

C toxins were separated using a 25 cm × 4.6 mm i.d., 5 µm Beta Basis C8 column (Thermo, Fisher Scientific, Madrid, Spain), with the column oven at 20 °C. Solvent A is 2 mM tetrabutyl ammonium phosphate aqueous solution adjusted to pH 5.8 using 1% NH_4_OH. Solvent B is 2 mM tetrabutyl ammonium phosphate in 4% MeCN, pH 5.8. The gradient used is 0% B in the first 8 minutes, 0%–100% B over the next 7 min, 100% B for one minute, 100%–0% B for 3 minutes and 0% B for 5 min before the next injection.

STX and GTX toxins group were separated on a 15 cm × 4.6 mm i.d., 3.5 µm Zorbax Bonus-RP column (Agilent Technologies, Madrid, Spain), with the column oven at 30 °C. Initially, solvent A was 8.25 mM heptane sulfonate, 5.5 mM H_3_PO_4_ aqueous solution adjusted to pH 7.1 using NH_4_OH 28%–30%. Solvent B was 8.25 mM heptane sulfonate, 16.5 mM H_3_PO_4_ in 11.5% MeCN, pH 7.1 using NH_4_OH 28%–30%. The gradient used in this case was 0% B over 8.4 min, 100% B at 8.5 min for 10 min, 0% B for 9 min before the next injection.

Injection volume for this method can be altered (10–30 µL for the GTX and STX toxins group and 5–20 µL for the C toxins) to improve detectability; in this case 10 and 5 µL were used for GTXs/STXs and Cs toxins respectively. The analysis time for some authors is 24 min [18] but it may vary slightly depending on how toxins elute; in this work the running time was 27 min for GTXs/STXs.

The flow rate was 0.8 mL/min, the column eluate is mixed into a T with the oxidant: 100 mM H_3_PO_4_, 5 mM H_5_IO_6_ aqueous solution adjusted to pH 7.8 with 5 M NaOH; the oxidant flow was 0.5 mL/min. The resulting mix is heated while passing through a homemade knitted teflon coil (5 m × 0.50 mm i.d.) immersed in a water bath at 80 °C. It was then acidified in another T with 0.1 M nitric acid at a flow rate of 0.3 mL/min, to reach a pH outflow ranging between 5–7 [26]. The fluorescent eluted derivatives were monitored using a fluorescence detector at 330 and 395 nm excitation/emission wavelengths, respectively. All mobile phases and postcolumn reagents must be filtered through a 0.2 µm nylon filter membrane before use. The liquid chromatography post-column oxidation system is the same described in PCOX method [18] except for the post-column reaction module that in this case is a water bath.

For the GTXs and STXs group, standards were diluted with 0.003 M HCl and for C toxins DIW (pH = 5) was the solvent, to have stock standard solutions with a range of concentrations from 7.9–28.6 µM. Working calibration solutions (0.32 µM to 2.66 µM) are prepared diluting stock standards in toxin-free shellfish tissue extract for GTXs and STXs toxins and in DIW (pH = 5) for Cs toxins. Stock and working solutions were stored at 4 °C and under −20 °C for GTXs and STXs group and Cs group respectively [27].

## 5. Conclusions

In conclusion, the behavior of toxins in different matrices is different. For mussels and clams, working conditions can be the same because the matrix behaves the same way. However, in scallops and oysters, interference from matrix peaks is significant and can lead to erroneous identification and quantification of the toxins, especially for GTX1, GTX4 and dcGTX3. Therefore, it is necessary to use different working conditions depending on the samples to be analyzed. Furthermore, it was found that different species as well as temporal and geographical variations will generate significant differences in the scallop matrix; however, in the case of clams, differences were observed only when samples with different geographical origins are compared.
